# Supplementary material for: Cryo-EM structure of the conjugation H-pilus reveals the cyclic nature of the TrhA pilin
Source: Proc Natl Acad Sci U S A. 2025 Apr 17;122(16):e2427228122. doi: 10.1073/pnas.2427228122 (PMC12037004; doi:10.1073/pnas.2427228122)
Supplement: Supplementary file 1 — Appendix 01 (PDF) [file pnas.2427228122.sapp.pdf]

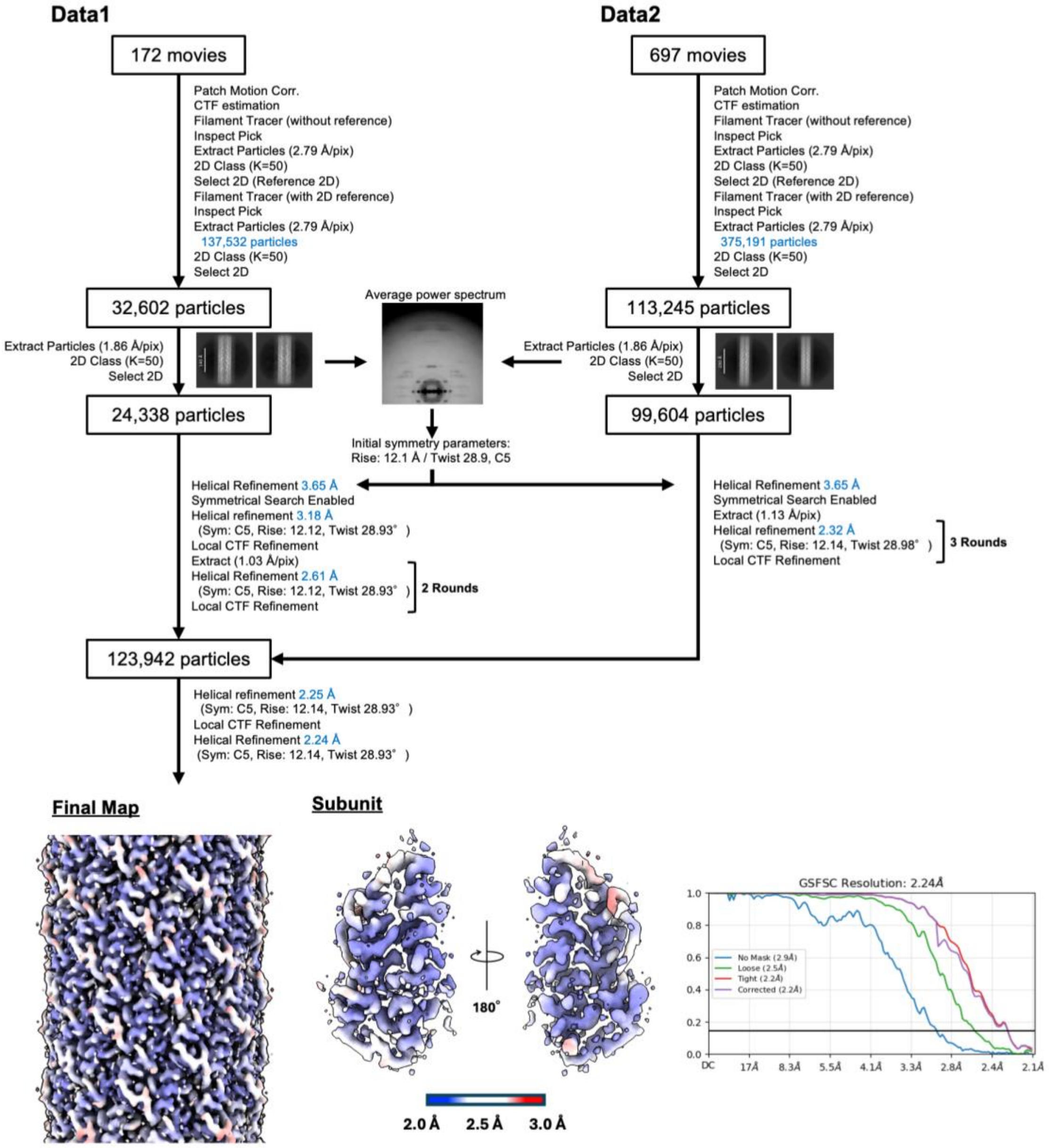

Supplementary Figure 1. Cryo-EM data processing workflow. Data processing workflow for H-pilus. All the processing was performed by cryoSPARC (v.4.6). The Final map was colored according to the local resolution. Gold-standard FSC curve of the final map was shown. The resolution was cut-off at FSC=0.143.

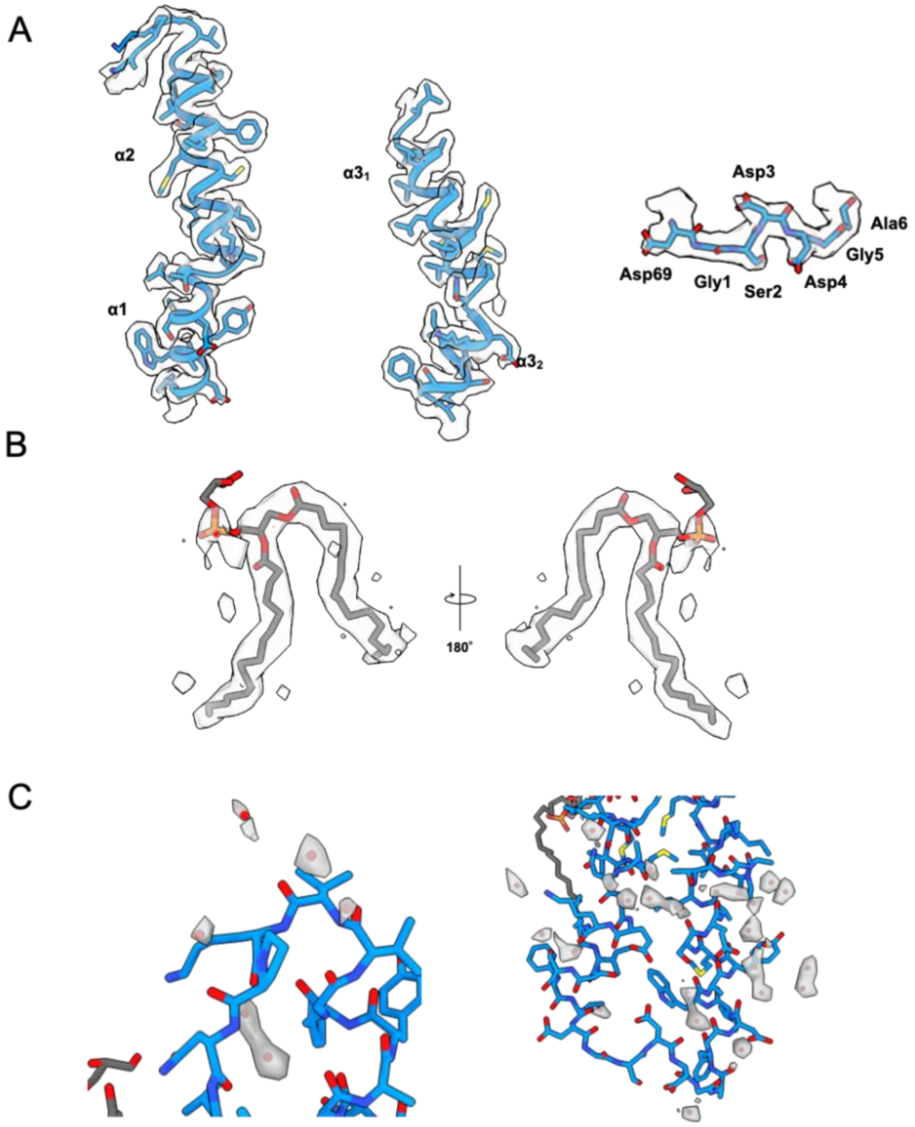

Supplementary Figure 2. Cryo-EM density map and fitting models. The cryoEM density map of covering (A) helix α1-3 and cyclisation position (B) lipid (C) water molecules.

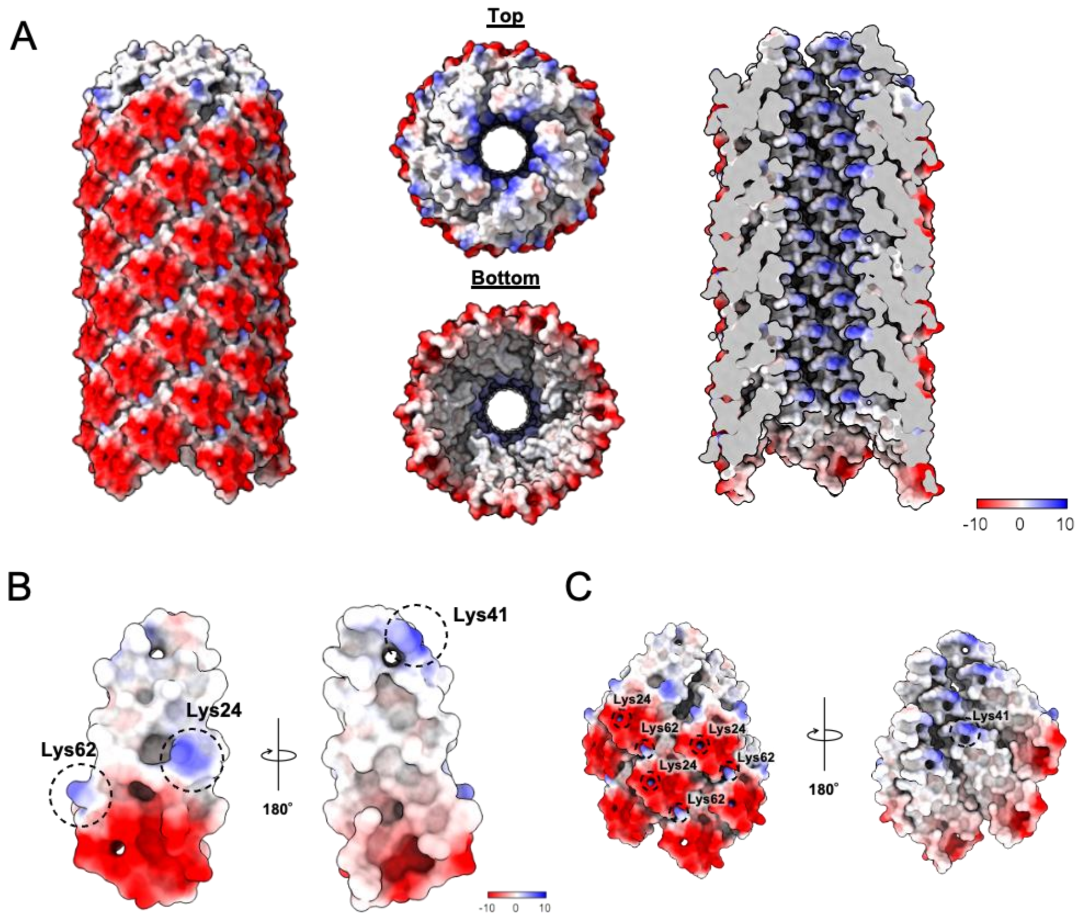

Supplementary Figure 3. The surface Electron charge analysis of H-pilus. (A) The surface electrostatic charge of H-pilus is shown in the side view (left), top and bottom views (middle), and slice view (right). The electrostatic potential was calculated in the absence of the lipid, and it is colored from red (negative) to white (neutral) to blue (positive). (B) A detailed surface electrostatic of the TrhA pilin is shown in two orientations. Key lysine residues (Lys24, Lys41, and Lys62) are highlighted with dashed circles, showing their positions in positively charged regions. (C) The neighboring subunits are shown, and analysis of electrostatic interactions between subunit and subunit. The view is shown in two orientations, highlighting key lysine residues.

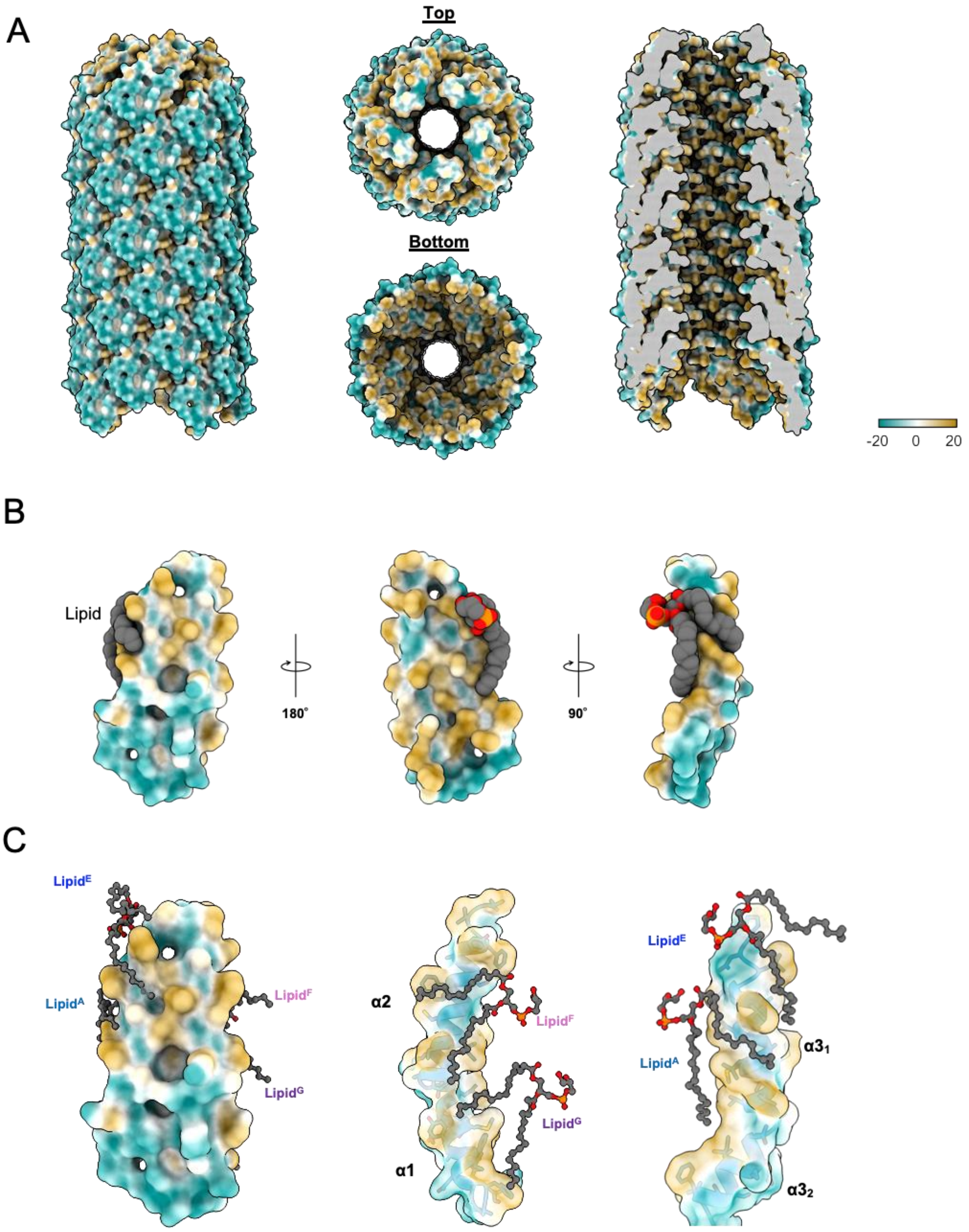

Supplementary Figure 4. The surface hydrophobicity analysis of H-pilus. (A) The Surface hydrophobicity analysis of H-pilus is shown in the side view (left), top and bottom views (middle), and slice view (right). The hydrophobic potential is coloured from yellow (hydrophobic) to white (neutral) to blue (hydrophilic). (B) A detailed surface hydrophobicity of the TrhA pilin and its association with the PG lipid is shown in three orientations. (C) Surface hydrophobicity of lipid-binding pockets in α1, α2, and α3.

Supplementary Table 1. Strains, plasmids and primers used in the generation of the MG1655 Δ*trp*, Δ*fimA* and R27 trhA mutants.

**Strain**	**Description**	**Resistance**	**Reference**
MG1655	*Escherichia coli* K12 strain (template for directed mutagenesis of *trp*- mutant)	-	Blattner *et al* 1997
CC118λpir	Triparental mating donor strain	-	Herrero *et al* 1990
E.coli pRK2013	Triparental mating helper strain	Kanamycin	Figurski *et al* 1970

**Plasmid**	**Description**	**Resistance**	
R27	IncH1 conjugative plasmid	Tetracycline	Alonso *et al* 2005
drR27	Derepressed R27 plasmid in which resistance was swapped from tetracycline to chloramphenicol	Chloramphenicol	Gibert *et al* 2020
pSEVA612S	Tri-parental mating mutagenesis vector	Gentamycin	Low *et al* 2019
pACBSR-Sm	Lambda red fragment expressing plasmid.	Streptomycin	Low *et al* 2019

**Primer**	**Sequence (5'→3')**	**Description**
pSEVA612s vector_fwd	tttccacggtTAGGGATAACAGGGTAATTAC	Amplifies linear pSEVA612S
pSEVA612s vector_rev	gcaacgcgcaTAGGGATAACAGGGTAATC	
down trp HR_fwd	gttatccctaTGCGCGTTGCGGATCATTTTTAAAATTAC	Amplifies downstream Homology region flanking *trp* operon
down trp HR_rev	gagaataacaTCCCACAGCCGCCAGTTC	
up trp HR_fwd	ggctgtgggaTGTTATTCTCTAATTTTGTTCAAAAAAAAG	Amplifies upstream homology region flanking *trp* operon
up trp HR_rev	gttatccctaACCGTGGAAATTTCCACG	
B.ISceI	TAGGGATAACAGGGTAAT	Amplifies linear pSEVA612S
fim HR1_fwd	GGATTACCCTGTTATCCCTAcatggcgtaagctgacgaatc	Amplifies upstream Homology region flanking *fim* operon
fim HR1_rev	agcaatgtcctgtgatttctcgtttttcccttataattacagacg	
fim HR2_fwd	gtaattataagggaaaaacgagaaatcacaggacattgctaatgctg	Amplifies downstream homology region flanking *fim* operon
trhA_fwd	gttatccctaagtgacttttacgataagc	Amplifies wild-type trhA
trhA_rev	gttatccctagctccatcaagaatagag	
Δ*trhA*_fwd	tttgttagaacaataacaatgaggg	Inverse PCR primers to delete the entire *trhA* open reading frame
Δ*trhA*_rev	aattactccgtgttgatttg	
*trhA*Δ64-74_fwd	aattactccgtgttgatttg	Inverse PCR primers to delete the amino acid residues 64-74 of mature TrhA
*trhA*Δ64-74_rev	aattactccgtgttgatttg	

Supplementary Table 2. Data collection and processing parameters, and refinement statistics.

	H-pilus
**PDB entry**	9HVC
**EMDB entry**	EMD-52431
**Data collection and processing**	
Magnification	130,000
Microscope	Titan Krios G2
Voltage (kV)	300
Detector	Falcon 4i
Electric exposure (e^-/Å)	50
Defocus range (µm)	-0.8 to -1.8
Pixel size (Å)	0.921
Data Processing Program	cryoSPARC (v.4.6.1)
Movies	869
Initial / Final particle images (no.)	492,091 / 123,942
Symmetry imposed	C5
Helical rise (Å)	12.14
Helical twist (°)	28.93
Map resolution (Å)	2.2
FSC threshold	0.143
**Refinement**	
Refinement Program	PHENIX (v.1.20.1)
Map:model resolution (Å)	2.6
FSC threshold	0.5
Model composition	
Non-hydrogen atoms	578
Protein residues	69
Water	31
R.m.s. deviations	
Bond length (Å)	0.003
Bond angles (°)	0.388
Validation	
MolProbity score	1.22
Clashscore	4.45
Ramachandran plot	
Favored / Allowed (%)	100 / 0
Disallowed (%)	0.00
Mask CC	0.88
